# Mutual regulation of PD-L1 immunosuppression between tumor-associated macrophages and tumor cells: a critical role for exosomes

**DOI:** 10.1186/s12964-024-01473-5

**Published:** 2024-01-09

**Authors:** Banglu Wang, Daoan Cheng, Danyu Ma, Rui Chen, Dong Li, Weiqing Zhao, Cheng Fang, Mei Ji

**Affiliations:** Departments of Oncology, The Third Affiliated Hospital of Soochow University, 185 Juqian Street, Changzhou, 213003 China

**Keywords:** Exosome, PD-L1, Tumor-associated macrophages, Immunosuppression

## Abstract

Tumor cells primarily employ the PD-1/PD-L1 pathway to thwart the anti-tumor capabilities of T lymphocytes, inducing immunosuppression. This occurs through the direct interaction of PD-L1 with PD-1 on T lymphocyte surfaces. Recent research focusing on the tumor microenvironment has illuminated the pivotal role of immune cells, particularly tumor-associated macrophages (TAMs), in facilitating PD-L1-mediated immunosuppression. Exosomes, characterized by their ability to convey information and be engulfed by cells, significantly contribute to promoting TAM involvement in establishing PD-L1-mediated immunosuppression within the tumor microenvironment. Exosomes, characterized by their ability to convey information and be engulfed by cells, significantly contribute to promoting TAM involvement in establishing PD-L1-mediated immunosuppression within the tumor microenvironment. In addition to receiving signals from tumor-derived exosomes that promote PD-L1 expression, TAMs also exert control over PD-L1 expression in tumor cells through the release of exosomes. This paper aims to summarize the mechanisms by which exosomes participate in this process, identify crucial factors that influence these mechanisms, and explore innovative strategies for inhibiting or reversing the tumor-promoting effects of TAMs by targeting exosomes.

## Introduction

Exosomes, which are nanoscale vesicles produced via exocytosis by both normal and tumor cells, play a critical role in mediating the interaction between tumor and immune cells within the tumor microenvironment [1]. They represent a distinct mode of signaling in addition to direct cell-to-cell communication and indirect communication through chemokines or cytokines [2, 3]. Exosomes contribute to the immunosuppressive characteristics observed in almost all lymphocyte and bone marrow cell populations, thereby assisting tumor cells in evading immune surveillance [4].

Tumor-associated macrophages (TAMs) are a type of immune cells present in the tumor microenvironment, primarily located in the tumor stroma where they regulate inflammatory responses. Recent evidence has highlighted that TAMs not only contribute to tumor progression but also play a role in immunosuppression [5, 6].

PD-1/PD-L1 signaling pathway serves as a critical checkpoint for tumor immune evasion [7]. Tumor-infiltrating T cells, influenced by the tumor microenvironment, exhibit high levels of PD-1 expression. However, their ability to kill tumor cells is significantly hindered upon interaction with PD-L1 expressed on the tumor surface [8].

Although immune checkpoint inhibitors have revolutionized cancer treatment, the response rate in patients receiving these therapies has not met expectations. This necessitates further exploration of the underlying mechanisms leading to resistance against immune-based therapies.

TAMs play a crucial role in mediating PD-1/PD-L1 immunosuppression, primarily through three mechanisms: exosome secretion, PD-L1 expression, and cytokine secretion [9]. This perspective highlights that TAMs actively contribute to the immune evasion of malignancies. Exosomes act as passive agents, implicating TAMs in the continuous invasion of tumors, and they are among several factors in the tumor microenvironment that influence the polarization of tumor-associated macrophages. Studying how exosomes promote the development of PD-1/PD-L1 immunosuppression between tumor and immune cells will help develop strategies to improve immunotherapy efficacy and overcome immune resistance. This paper reviews the recent progress in this field and its potential mechanisms. Experimental evidence supports exosomal targeting to enhance immune effectiveness, emphasizing the importance of exosomal targeting as a key strategy to combat immune resistance.

## Exosomes regulate the pro-tumor and anti-tumor state of TAMs

In a broader context, TAMs do not represent a specific type of macrophage, but rather a heterogeneous population of macrophages with diverse molecular expressions and functional states infiltrating the tumor microenvironment. They can be roughly categorized into anti-tumor M1 type and pro-tumor M2 type, although TAMs predominantly exhibit an M2 phenotype [10]. M1 macrophages are characterized by the expression of molecules such as CD86 and possess pro-inflammatory functions [11]. On the other hand, M2 macrophages, which have high IL-10 expression and low IL-12 expression, can be further subdivided into different subtypes (M2a, M2b, M2c, M2d) with unique combinations of molecular markers that support the malignant behavior of most tumors [12]. It is worth noting that the polarization state of TAM and the ratio of different subtypes change dynamically with specific time, which is related to the complex stimulus signals in the tumor microenvironment [13]. Additionally, intermediate cells between the two extreme M1 and M2 markers may simultaneously express markers associated with both M1 and M2 phenotypes [14].

Tumor-derived exosomes facilitate macrophage recruitment and have been shown to influence the polarization of M1/M2 type macrophages in various tumor types [15–17]. Exosomes play a significant regulatory role in promoting the pro-tumor phenotype of macrophages by carrying nucleic acids, proteins, lipids, and other important regulatory substances [18]. For instance, the incorporation of MiR-3591-3p into glioma exosomes promotes M2-type polarization of macrophages through the JAK2/PI3K/Akt/mTOR and STAT3 pathways, thereby enhancing tumor invasion and migration [19]. Gallbladder cancer cells effectively utilize exosomes to transport leptin to macrophages, thereby influencing M2 macrophage polarization. Inhibiting leptin reverses this process’s pro-tumor growth effect [20]. By controlling macrophage polarization via exosomes, tumors facilitate the development of a pre-metastatic niche. Researchers discovered that the polarization of pulmonary macrophages by Caveolin-1 transported by exosomes could be inextricably linked to pre-metastatic microenvironment creation before breast cancer (BC) cells metastasize to the lung [21]. Tumor cell-produced microRNA (miR)-21 and miR29a exosomes stimulate the production of Toll-like receptor (TLR)-mediated pro-inflammatory proteins in macrophages, which are linked to NF-κB activation and collectively implicated in the pre-tumor inflammatory phase [22]. In addition to promoting malignant tumor growth and distant spread, M2 macrophages polarized by tumor-derived exosomes also contribute to reducing tumor treatment sensitivity. For instance, non-small cell lung cancer induces macrophage M2 polarization by releasing phosphoribosyl pyrophosphate synthetases 2 exosomes, thereby increasing the cisplatin toxicity threshold for tumor cells [23]. The direction of TAMs polarization is also influenced by exosomes produced by different cell types in the tumor microenvironment. Cancer-associated fibroblasts educate monocytes to differentiate into the M2 macrophage phenotype, which supports BC growth. Exosomes produced by this phenotype also exhibit high expression of the highly invasive miR-181a. Furthermore, it has been demonstrated that miR-320a, released by fibroblasts, controls the direction of M2-type polarization of macrophages [24]. The progression of prostate cancer has also been shown to be related to the regulation of the M2-type polarization direction of macrophages by miR-320a secreted by fibroblasts [25].

## TAM-derived exosomes promote tumor growth

Exosomes serve as a critical medium for communication between TAMs and tumor cells within the tumor microenvironment. TAM-derived exosomes play a significant role in influencing tumor formation through various mechanisms, highlighting the importance of their components [26, 27]. These exosomes derived from TAMs contribute to the continued malignant growth of tumors by regulating tumor cell stemness, promoting tumor angiogenesis, facilitating tumor metastasis, and inducing treatment resistance [28–31]. Importantly, TAM-derived exosomes also contribute to the resistance of tumor cells towards targeted drugs, in addition to chemotherapy drugs [32, 33]. Furthermore, TAM-derived exosomes play a crucial role in metabolic reprogramming. For instance, exosomes containing long non-coding RNA derived from TAMs activate glycolysis in hepatocellular carcinoma (HCC) via the miR-548 s/ALDH1A3 pathway, demonstrating that metabolic reprogramming is an essential mechanism through which exosomes promote malignant proliferation [34].

Several factors, including hypoxia [35], epithelial-mesenchymal transition [36], gene mutations [37], and various signaling pathways [38], influence TAMs mediated by exosomes that promote tumor growth. Hypoxia has long been recognized as a critical physiological component in the tumor microenvironment. Tumor-derived exosomes, which are released at higher levels and exhibit changes in heterogeneity under hypoxic conditions, play a role in transferring hypoxic information within the tumor microenvironment. These exosomes possess distinct characteristics in terms of cargo content, cellular recognition, and internalization by target cells [39, 40]. Exosomes facilitate the interaction between TAMs and tumor cells or other immune cells in the tumor microenvironment, thereby promoting tumor growth and invasion.

## Exosomes mediate the regulation of PD-L1 expression by TAMs

**Fig. 1:**
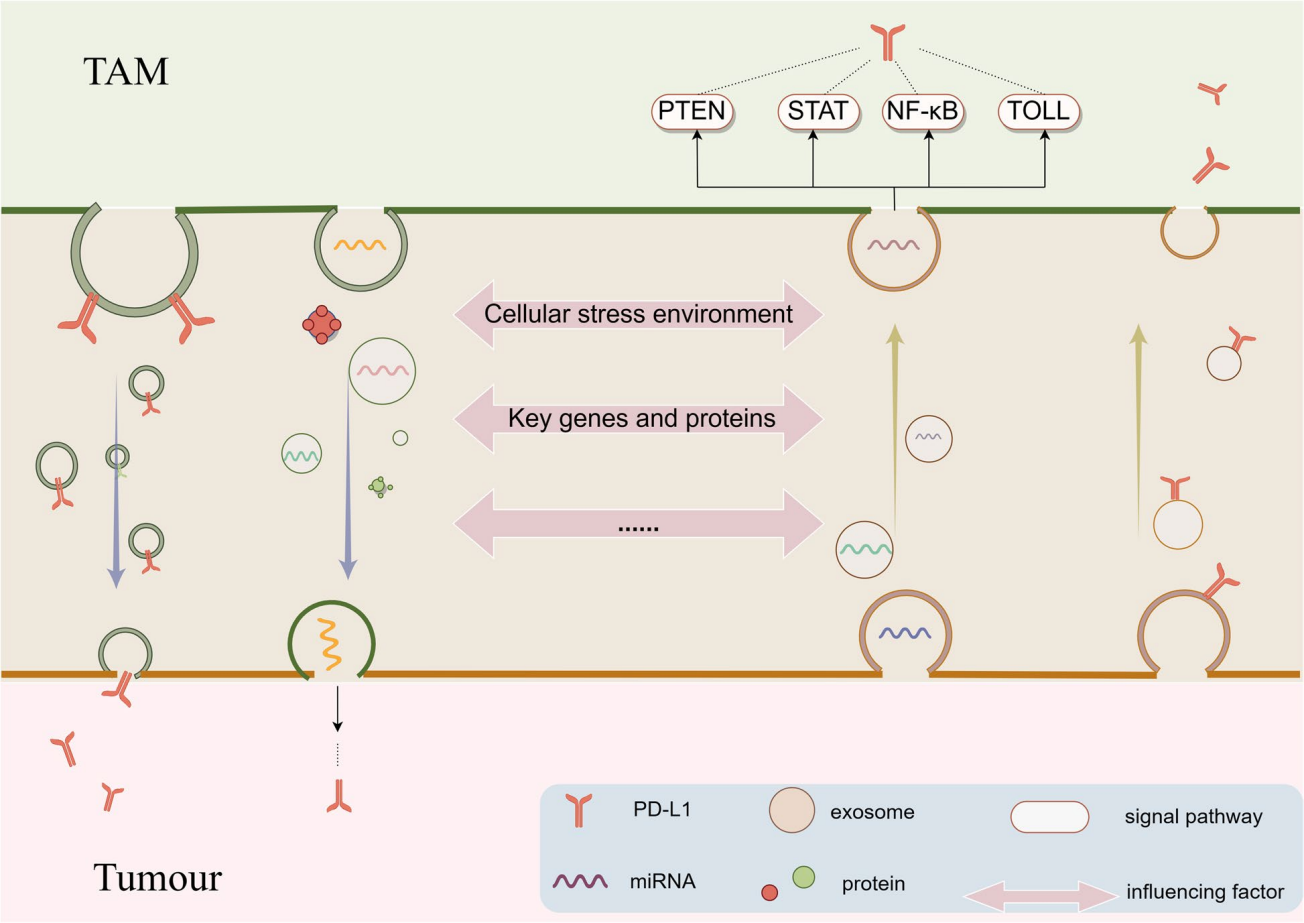
Mechanism of exosomes mediated TAM regulation of PD-L1 expression. Exosomes regulate the expression of tumor cells and TAM PD-L1 mainly through two ways: (**a**) Direct delivery dependent on PD-L1; (**b**) By acting on PTEN, STAT, TOLL receptor, NF-κB mediated signaling pathways

Yang et al.’s research has provided long-standing evidence that PD-L1 is present simultaneously in tumor cells and the exosomes they produce [41]. PD-L1 exosomes produced by tumor cells enhance the immunosuppressive ability of the tumor microenvironment by directly binding with T cells and indirectly binding with TLRs on myeloid cells [42]. Exosomes carrying PD-L1 compromise the body’s ability to fight tumors by transmitting PD-L1 to other cells in the tumor microenvironment, such as macrophages, actively suppressing T cells’ immunological functions [41]. Phagocytes, including macrophages, have a more professional approach to phagocytosis than non-phagocytic cells and absorb PD-L1 exosomes in the tumor microenvironment to escape immune surveillance [41, 43]. Gastric cancer (GC)-derived exosomes cause macrophages to polarize towards the M2 type and express PD-1, leading to increased production of IL-10 and accelerated tumor growth by preventing T cell activation [44]. Gu and his colleagues found that miR-92a-3p exosomes from GC can also be ingested by pulmonary macrophages, activating the ERK signaling pathway by inhibiting the expression of PTEN in macrophages and promoting the expression of PD-L1 in macrophages through transcriptional regulation [45]. Similarly, exosomes from TAMs also regulate the expression of PD-L1 in tumor cells. M2-type TAMs use exosomes to enhance PD-L1 expression in GC cells [46]. Conversely, M1 macrophages downregulate PD-L1 expression in GC cells by loading miR-16-5p exosomes [47]. Crosstalk between different immune cell types in the tumor microenvironment also plays an important role in promoting the expression of PD-L1 on TAMs. Mesenchymal stem cells produce exosomes carrying TGF-β, C1q, and signaling proteins, which induce overexpression of PD-L1 in undifferentiated monocytes and macrophages **(**Fig. 1**)** [48].

### Mechanism of exosomes mediated TAM regulation of PD-L1 expression

#### PTEN/PD-L1 axis

Strong evidence has linked the deletion of the PTEN gene to tumor PD-L1 expression in various types of cancer [49–51]. Breast cancer (BC) cells increase PD-L1 expression in macrophages to evade the immunological response, cooperating with cancer cells through a PTEN-mediated signaling pathway. Exosomes generated from BC cells are enriched in miR-27a-3p, contributing to this process [52]. Similarly, miR-23a-3p, abundant in exosomes produced from hepatocellular carcinoma (HCC), reduces the expression of PD-L1 in TAMs through PTEN-mediated signaling pathways [53]. When comparing miR-183-5p exosomes produced by intrahepatic cholangiocarcinoma cells with primary human intrahepatic bile duct epithelial cells, higher expression was observed in the HCC. This encourages the activation of PD-L1 in macrophages through the PTEN/AKT/PD-L1 signaling pathway, enhancing tumor immune evasion and reducing patient survival [54]. Surprisingly, TAMs possess higher levels of PD-L1 than colorectal cancer (CRC) cells, and CRC cells stimulate PD-L1 expression in macrophages by controlling the PTEN/AKT pathway via miR-21-5P exosomes (Table 1) [55].

**Table 1:** Exosomes that regulate PD-L1 immunosuppression

Axis	Exosome cargo	Generate from	Signaling pathway	Action on	Reference
PTEN/PD-L1	miR-92a-3p	GC	miR-92a-3p-PTEN-EPK-PD-L1	TAM	[45]
	miR-183-5p	intrahepatic cholangiocarcinoma	miR-183-5p-PTEN-AKT-PD-L1	TAM	[54]
	miR-27a-3p	BC	miR-27a-3p- MAGI2- PTEN-PI3K-AKT- PD-L1	TAM	[52]
	miR-23a-3p	HCC	miR-23a-3p- PTEN- AKT	TAM	[53]
	miR-21-5p/ miR-200a	CRC	miR-21-5p/ miR-200a- PTEN-AKT-PD-L1	TAM	[55]
STAT/PD-L1	LOXL4	HCC	LOXL4- IFN- STATs- PD-L1	TAM	[56]
	miR-21-5p/ miR-200a	CRC	miR-21-5p/ miR-200a- SCOC1-STAT1-PD-L1	TAM	[55]
	/	Laryngeal carcinoma	STAT3- PD-L1	TAM	[57]
	/	glioblastoma-derived stem cells	p-STAT3 and/or p44/42 MAPK (Erk1/2)- PD-L1	TAM	[58]
	/	HCC	STAT3-PD-L1	TAM	[59]
	/	/	TLR4-MyD88-p38-STAT3-PD-L1	TAM	[60]
	hca-circRNA-001264	AML	hca-circRNA-001264- RAF1- p38- STAT3-PD-L1	TAM	[61]
TOLL/PD-L1	/	LUAD	TLR2- NF-kB- PD-L1	TAM	[62]
	/	/	TLR4-MyD88-p38-STAT3-PD-L1	TAM	[60]
	hY4	CLL	hY4- TLR7- PD-L1	TAM	[63]
NF-κB/PD-L1	/	LUAD	TLR2- NF-kB- PD-L1	TAM	[62]
Others	/	TAM	P38MAPK- PD-L1	GC	[46]
	IL-32γ	MM	PR3-PFKFB3-JAK1-PD-L1	TAM	[64]

Abbreviations: *LOXL4* Lysyl oxidase-like 4, *GC* gastric cancer, *BC* breast cancer, *HCC* hepatocellular carcinoma, *CRC* colorectal cancer, *AML* acute myeloid leukemia, *LUAD* lung adenocarcinoma cell, *CLL* Chronic lymphocytic leukemia, *MM* multiple myeloma, *TLR* Toll-like receptor

#### STAT/PD-L1 axis

The Signal Transducer and Activator of Transcription (STAT) family, consisting of numerous members, exerts a significant regulatory influence on tumor proliferation, immune evasion, and metastasis [65]. Loading exosomes with lysyl oxidase-like 4 (LOXL4) produced by liver cancer cells promotes macrophage PD-L1 expression, mediated by interferon and STATs [56]. Microparticles loaded with PD-L1 in triple-negative breast cancer stimulate the TBK1/STAT6 signaling pathway, polarizing internalized macrophages into an immunosuppressive phenotype and promoting the establishment of immunosuppressive features in the tumor microenvironment [66]. Macrophage-derived exosomes promote PD-L1 expression in laryngeal squamous cell carcinoma through the STAT3 transcription pathway, which was further confirmed by the addition of STAT3 inhibitors [57]. Glioblastoma plays a crucial role in inducing the formation of an immunosuppressive phenotype in macrophages through the STAT3-mediated signaling pathway, facilitating dynamic communication between malignant tumor cells [58]. Melatonin-exposed HCC cells reduce PD-L1 expression in macrophages via the STAT3 pathway mediated by exosomes, thereby altering the characteristics of exosomes initially promoting liver tumor growth [59]. Exosomes from CRC carrying miR-200a and miR-21-5p also stimulate the STAT1 pathway within the macrophage, which is crucial in encouraging PD-L1 expression [55].

#### TOLL/PD-L1 axis

The intricate network of TLR-mediated signaling pathways is a vital component in the complex landscape of cancer development. As a pattern recognition receptor, TLRs serve as the crucial link between innate and adaptive immunity, allowing for effective immune responses against invading pathogens and malignant cells alike [67]. The expression of PD-L1 in M2 macrophages through TLR4-dependent signaling effectively shapes the immunosuppressive phenotype of macrophages, preventing T cell proliferation and providing opportunities for tumor immune escape. These secretory autophagosomes, derived from malignant tumors, are found in the pleural and abdominal fluids of tumors [60]. Exosomes secreted by non-small cell lung cancer have an immunosuppressive effect on macrophages, dependent on PD-L1 activation via TLR2 signaling [62]. Exosomes produced by tumor cells in chronic lymphocytic leukemia activate TLR7 to mediate PD-L1 production in monocytes and release cytokines that stimulate tumor growth [63].

#### NF-κB/PD-L1 axis

NF-κB has long been recognized as a key player in inflammatory-mediated tumorigenesis [68], and accumulating evidence suggests that activated NF-κB is a key regulator of different functional states of TAMs and can directly mediate TAM PD-L1 expression [69]. Morrissey et al. also emphasized the role of NF-κB-dependent pathways in the action of tumor-derived exosomes on TAMs, which, in addition to Toll-like receptors, mediate enhanced glycolysis and lactate production through independent activation of NOS2 and HIF-1α, thereby promoting M2-type macrophage polarization. Increased lactate-driven de novo synthesis of PD-L1 expression is observed in TAMs, which mediates pre-metastatic niche formation; this effect is unrelated to exosome PD-L1 translocation [62].

### Factors affecting exosomes-mediated TAMs regulation of PD-L1-mediated immunosuppression

#### Cellular stress environment

The ER stress marker was closely and positively related to the expression of M2 macrophages PD-L1 in oral squamous cell carcinoma. In vivo and in vitro experiments revealed that co-culturing with tumor cells subjected to ER stress could promote PD-L1 expression in macrophages [70]. Similarly, ER stress was found to upregulate PD-L1 expression in macrophages in breast cancer (BC) and hepatocellular carcinoma (HCC) tissues through exosomes [52, 53]. Reactive oxygen species (ROS) generated by redox reactions in the tumor immune microenvironment played a significant role in regulating PD-L1 on macrophages. Elevated reactive oxygen species (ROS) downregulated exosomal miR-155-5p expression in tumor cells, impacting PD-L1 expression. Neutralization with N-acetyl-L-cysteine (NAC) restored miR-155-5p levels, reducing macrophage migration and tumor infiltration while enhancing CD8 T cell function [71]. Tumor-derived exosomes were shown to produce lactate, which played a crucial role in metabolic reprogramming and increased macrophage PD-L1 expression through NF-κB signaling [62]. The immunosuppressive effects of adenosine in HCC were specific. Exosomes from CD73-expressing liver cancer cells (circTMEM181 exosomes) induce macrophages to upregulate CD39 expression, leading to adenosine accumulation and changes in the immune microenvironment, forming resistance to anti-PD-1 drugs (Table 2) [75].

**Table 2:** Factors affecting the regulation of PD-L1 expression exosomes

Influencing factors	tumour	function	References
ER stress	OSCC	Up	[70]
ER stress.	BC	Up	[52]
ER stress	HCC	Up	[53]
oxygen species	ovarian cancer	Up	[71]
lactate	NSCLC	Up	[62]
H_2_O_2_	HCC	Up	[56]
xCT	melanoma	Down	[72]
GOLM1	HCC	Up	[73]
mechanical forces	BC	Up	[74]
melatonin	HCC	Down	[59]

Abbreviations: *ER* endoplasmic reticulum, *H2O2* hydrogen peroxide, *xCT* cystine-glutamate exchange, *GOLM1* Golgi membrane protein 1, *OSCC* Oral squamous cell carcinoma, *NSCLC* non-small-cell lung cancer, *HCC* hepatocellular carcinoma

#### Key genes and proteins

Golgi apparatus protein 1 was found to transport PD-L1 protein and upregulate PD-L1 expression in macrophages in hepatocellular carcinoma (HCC), thus affecting the response of liver cancer to anti-PD-L1 therapy. This process depended on proteasomes to reduce PD-L1 protein consumption and promote PD-L1 extracellular transport by inhibiting Rab27b. Treatment with proteasome inhibitors reduced PD-L1 expression, providing evidence for the critical role of Golgi protein 1 in promoting the formation of tumor immunosuppressive environments [73]. CMTM6, an essential protein regulating PD-L1 expression, has gained unprecedented attention [76]. Pang et al. reported that CMTM6 was closely associated with macrophage infiltration and PD-L1 expression in oral squamous cell carcinoma. Transfer of CMTM6 to macrophages through exosomes promoted ERK1/2-mediated M2 polarization [77]. The expression of CMTM6 and PD-L1 in colorectal cancer (CRC) with mismatch repair defect was positively correlated with the density of M2 macrophages. The expression of CMTM6 in M2 macrophages was the best biomarker for predicting the effectiveness of PD-1/PD-L1 inhibitors in CRC patients [78].

#### Others

Sulfasalazine has been found to disrupt the redox balance of tumor cells, leading to increased production of reactive oxygen species and inhibition of tumor progression. In melanoma, sulfasalazine inhibits cystine-glutamate exchange, which promotes the production of two transcription factors, IRF4 and EGR1, that regulate PD-L1 expression. This enhances the secretion of PD-L1 exosomes and induces M2 polarization in macrophages, reducing sensitivity to immune checkpoint blockade (ICB) treatment [72]. Counteracting the effect of LOXL4 exosomes on increasing macrophage PD-L1 expression in hepatocellular cancer can be achieved by using copper or hydrogen peroxide scavengers [56]. In vitro experiments simulating the mechanical strain experienced by tumor cells during growth have shown that it promotes the release of PD-L1 exosomes from breast cancer cells in the tumor microenvironment, which are then internalized by macrophages [74]. Patients infected with hepatitis B virus produce exosomes containing viral DNA and proteins, which upregulate PD-L1 expression in endocytic monocytes/macrophages, contributing to immune suppression [79]. Melatonin, known for its immune regulatory effects, has been utilized in tumor treatment. In hepatocellular carcinoma cells, melatonin therapy reverses the downregulation of macrophage PD-L1 expression through the inhibition of the STAT3-mediated signaling pathway [59].. Researchers studying non-small cell lung cancer patients with varying degrees of obstructive sleep apnea have found that intermittent hypoxia induces hypoxia-inducible factor-1, which enhances macrophage PD-L1 expression induced by exosomes [80].

## Discussion

Tumors have the tendency and capability to regulate the expression of PD-L1 in tumor-associated macrophages (TAMs). TAMs undergo a transformation into tumor-promoting phenotypes after being influenced by exosomes released by tumor cells. This transformation then establishes a positive feedback loop through exosomes, leading to increased PD-L1 expression in tumor cells. This article reviews the mechanism of exosomes regulating PD-L1 expression between tumor-associated macrophages (TAM) and tumor cells. It has been observed that PTEN, STAT, TLR and NF-κB have the highest frequency of occurrence in different types of tumor signaling pathways, presenting potential targets for inhibiting TAMs’ regulation of the PD-1/PD-L1 immunosuppressive pathway. The specificity of exosomes is determined by various factors such as cell type, cellular state, and environmental factors including mechanical stress, ER stress, reactive oxygen species, adenosine, lactic acid, among others. Proteins like Rab27, TP53, and CMTM6 play key roles in regulating the formation of exosomes that carry information involved in PD-L1 expression regulation. Factors like melatonin, sulfasalazine, hepatitis B virus, and obstructive sleep apnea also influence the level of PD-L1 expression regulated by TAMs through exosomes to varying degrees. These findings provide valuable research directions for targeting exosome-mediated PD-L1 expression and blocking the immunosuppressive effect of TAMs on tumor PD-L1.

Targeting TAMs to enhance the efficacy of immunotherapy has been clinically validated through two main strategies: TAMs consumption and TAMs reeducation. Due to the unique biological characteristics of exosomes, which are easily customizable and carry substances, targeting or modifying exosomes has emerged as a novel approach to inhibiting the M2 polarization of TAMs, achieving TAMs reeducation, and improving the effectiveness of tumor treatment [81–83]. Exosomes can overcome the toxicity of nanoparticles and limitations of phagocytic clearance by targeting specific cargoes to lesions and tissues through cellular phagocytosis and homing mechanisms [84]. CpGODN, a tumor vaccine modified by apoptotic bodies, not only polarizes M1 macrophages to produce more tumor suppressor factors but also enables these polarized macrophages to infect nearby macrophages, leading to a cascade reaction of tumor inhibitory effects [85]. Exosomes play a crucial role in controlling macrophage dynamic polarization in vivo, allowing them to transition from a pro-tumor phenotype to an anti-tumor one [86]. Exosomes are engineered and modified to deliver functional molecules that re-educate TAMs [87–89]. Researchers often target microRNA and chemotherapy drugs for exosome payloads, and antisense oligonucleotides targeting key transcription factors and inhibitors targeting enzymes are also being considered [90–93]. Transfection of genes expressing pigment epithelial-derived factors into parental cells resulted in the ability of derived exosomes to promote the repolarization of macrophages from the M2 phenotype to the M1 phenotype [94]. Ovarian cancer cells overexpressing ETS1 secrete exosomes that are more easily internalized by macrophages, promoting M2 polarization to facilitate tumor cell metastasis to the omentum. Targeting Integrin αVβ5, which plays a crucial role in inhibiting the polarization process, with cilengitide weakens the driving effect of these exosomes [95].

It is noteworthy that engineering modifications of extracellular vesicles derived from tumor-associated macrophages (TAMs) offer several advantages in tumor treatment compared to vesicles derived from other cell types [96, 97]. The investigation into the modification of exosomes derived from tumor-associated macrophages (TAMs) to enhance the effectiveness of drug delivery is experiencing rapid growth, particularly in the context of M1 subtype, showcasing notable efficacy in reversing tumor chemoresistance [98–101]. For instance, Choo et al. successfully repolarized M2 TAMs into M1 macrophages using exosomes derived from macrophages, thereby enhancing the anti-tumor efficacy of anti-PD-L1 therapy [102]. By carrying anti-PD-L1 nanobodies or binding with anti-CD73 antibodies, exosomes derived from M1 TAMs acquire the ability to reverse tumor immune suppression and increase the sensitivity of tumor cells to radiation therapy [103, 104]. Hybrid cells combining macrophages and tumor cells can produce exosomes that effectively activate T cells to kill tumor cells, combining the immune-stimulating abilities of macrophages with the “homing ability” of circulating tumor cells. When combined with anti-PD-L1 therapy, these hybrid exosomes inhibit tumor progression and provide insights into personalized tumor immunotherapy [105]. Additionally, it is worth mentioning that Artemisia-derived nanovesicles have been found to enhance the effectiveness of PD-L1 immune checkpoint inhibitors, suggesting that cross-species approaches with common biological characteristics hold promise for tumor treatment [106].

The clinical application of PD-L1/PD-1 immune checkpoint inhibitors has transformed the treatment of advanced unresectable tumors, surpassing traditional radiotherapy and chemotherapy. However, immunotherapy still poses challenges, such as low response rates and drug resistance. Therefore, researchers must focus on screening patients who respond effectively to immunotherapy and developing strategies to improve and maintain drug efficacy. In recent years, the discovery of the tumor-promoting effect of immune cells in the tumor microenvironment has provided important clues for understanding tumor progression. To further identify and expand the population that responds to immune therapy, it is necessary to conduct more in-depth research on the mechanism of tumor immune suppression mediated by TAMs, providing more diverse treatment options. TAMs are among the most abundant immune cells in the tumor microenvironment, and their dynamic polarization and phagocytosis suggest that exosomes play a crucial role in TAM-mediated tumor PD-L1/PD-1 immunosuppression. The evolving understanding of exosomes, from metabolic waste transporters to important communication mediators between cells, has deepened research in the field of cancer. It is anticipated that exosomes will significantly contribute to suppressing TAMs and enhancing PD-L1 immune development.

## Data Availability

Not applicable.

## Abbreviations

PD-1: Programmed cell death protein 1
PD-L1: Programmed cell death ligand 1
TAM: Tumor-associated macrophages
MiR: MicroRNA
BC: Breast cancer
TLR: Toll-like receptor
STAT: Signal transformer and activator of the transcription
HCC: Hepatocellular carcinoma
ER: Endoplasmic reticulum
LOXL4: Lysyl oxidase-like 4
CRC: Colorectal cancer
